# Increased Choroidal Thickness in Keratoconus Patients: Perspectives in the Disease Pathophysiology

**DOI:** 10.1155/2019/2453931

**Published:** 2019-12-03

**Authors:** João Pinheiro-Costa, João Viana Pinto, Sara Perestrelo, João Nuno Beato, Luís Torrão, Elisete Brandão, Ângela Carneiro, Maria Dulce Madeira, Fernando Falcão-Reis

**Affiliations:** ^1^Department of Ophthalmology, Centro Hospitalar Universitário São João, Porto, Portugal; ^2^Department of Biomedicine, Faculty of Medicine, University of Porto, Porto, Portugal; ^3^Faculty of Medicine, University of Porto, Porto, Portugal; ^4^Department of Surgery and Physiology, Faculty of Medicine, University of Porto, Porto, Portugal

## Abstract

**Purpose:**

To analyze and compare choroidal thickness between keratoconus (KC) patients and age-matched non-KC subjects.

**Methods:**

A cross-sectional, case-control study. One hundred and thirty-four keratoconic eyes and 78 control eyes, from individuals aged from 12 to 30 years old, were studied. Patients with KC followed in Corneal Department of Centro Hospitalar São João, Porto, Portugal, were identified and consecutively included between December 2017 and February 2018. A spectral-domain optical coherence tomography (OCT) using depth enhanced imaging was performed, and choroidal thickness in the center of the fovea and at 500 *μ*m intervals along a horizontal section was measured and compared.

**Results:**

The statistical analysis showed that keratoconic eyes present a thicker choroid in every measured location (*p* < 0.05). Mean subfoveal choroidal thickness (SFCT) values obtained were 375.86 ± 89.29 and 322.91 ± 85.14 in keratoconus and control groups, respectively (*p* < 0.001). In a multivariate analysis, SFCT was significantly associated with spherical equivalent (*p*=0.004) and the presence of keratoconus (*p* < 0.001), but not with age (*p*=0.167), gender (*p*=0.579), or best-corrected visual acuity (*p*=0.178). In a “fixed model,” keratoconus patients were found to have a 67.55 *μ*m (95% CI 36.61–98.49) thicker subfoveal choroid compared to controls.

**Conclusion:**

Keratoconus patients seem to have a thicker choroid than healthy individuals. The exact pathophysiological mechanism resulting in a thicker choroid in KC patients is not known, but it could possibly be associated with inflammatory choroidal mechanisms.

## 1. Introduction

Keratoconus (KC) has been classically defined as a progressive, bilateral, and asymmetrical noninflammatory corneal ectasia. It is characterized by a progressive stromal thinning and several structural changes in the cornea, leading to its conical shape, decreased biomechanical strength, and scarring [1, 2]. Clinically, patients present irregular astigmatism and myopia with loss of visual acuity, photophobia, monocular diplopia, and glare. These symptoms have a strong impact on patients' quality of life, making this disease a public health problem with an economic burden associated with its management [3].

Recent epidemiologic studies determined an annual incidence of KC of 1 : 7.500 from the ages of 10 to 40 with an estimated prevalence of 265 cases per 100,000 in the general population [4]. Nevertheless, the prevalence of KC may vary according to ethnicity and region, but no gender predilection has been consistently reported [5].

Both genetic and environmental factors may contribute to the pathogenesis of KC [6]. The major environmental and behavior risk factors associated with the disease seem to be eye rubbing, contact lens wear, and ultraviolet light, which results in an increased oxidative stress in the cornea [7], whereas atopy, by itself, has shown some contradictory results [8–10]. Although the majority of KC cases are sporadic, there is a widespread concern about the genetically predisposition for KC. Recent studies suggest a complex trait involving multiple genes with variable penetrance [11].

KC has been classically defined as a noninflammatory disease since there is no evidence of inflammatory cell infiltration or neovascularization in the cornea [1, 2]. However, a large number of studies suggest a role for inflammation in the pathophysiology of this disease [12, 13]. A disrupted balance between proinflammatory and anti-inflammatory mediators in the tears of keratoconus patients has been reported [13, 14]. Increased levels of several inflammatory mediators (IL-1, IL-6, TNF-a, and MMP-9) have been consistently found in the tears of KC patients [15–17]. These inflammatory mediators operate actively at the ocular surface and affect the corneal microenvironment, which could play an important role in the KC pathogenesis [12, 13]. In addition to local activation of inflammatory pathways, there is accumulating evidence that systemic inflammatory changes and systemic oxidative stress may affect the corneal microenvironment in KC [12, 18, 19]. Moreover, Shetty et al. showed that treatment of KC patients with cyclosporine A for 6 months reduced tear matrix metalloproteinase-9 (MMP-9) levels and led to a local reduction in corneal curvatures determined by corneal topography maps [20].

The choroid has been implicated in the pathogenesis of many inflammatory disorders of the eye. Being both the most vascularized tissue of the eye and the tissue with the highest blood flow per weight unit, choroid may play a role in the pathogenesis of ocular inflammatory processes. Thickening of the choroid, possibly caused by an infiltration of the choroid by inflammatory cells, has been demonstrated in the active phases of Vogt–Koyanagi–Harada disease (VKH), Behçet's disease, sympathetic ophthalmia, and posterior scleritis [21–25]. Moreover, choroidal thickness seems to be subclinically involved in some systemic inflammatory diseases without ophthalmologic manifestations [26].

In recently published data, two studies showed an increased choroidal thickness profile in KC patients. Gutierrez-Bonet et al. with a swept-source (SS) optical coherence tomography (OCT) showed that CT in KC patients is statistically thicker than in healthy population, with an average increase of 34% [27], and Akkaya with a spectral-domain (SD) OCT showed similar results [28]. These studies described for the first time changes in the choroidal profile of KC patients, a disease that was considered purely corneal until then [27]. On the other hand, Yilmaz et al. did not find any differences in the choroidal profile of pediatric KC patients in a controlled study of patients with a mean age of 12 years [29].

The SD-OCT is a noninvasive imaging modality that provides in vivo characterization of three-dimensional retinal anatomy. With enhanced depth imaging (EDI) software integrated into SD-OCT devices, it is possible to visualize the entire choroid with high resolution. Thus, choroidal morphology and especially small changes in choroidal thickness have been assessed using this technology in several pathological and physiological conditions. Although the measurement of choroidal thickness is currently made manually, the intervisit, interobserver, and intersystem agreement is very good and the measurements are very reliable [30].

Based on these different studies, the authors compared choroidal morphology and thickness in keratoconic eyes with age-matched controls and discussed the possible implication of these findings in the pathophysiology of the disease.

## 2. Materials and Methods

### 2.1. Patient Selection and Data

We have designed a cross-sectional, case-control study in which 74 patients with KC and 39 control subjects, without clinical or topographic KC signs, were enrolled. This study was approved by the ethics committee of Centro Hospitalar São João, fulfilled all requirements of the Declaration of Helsinki, and was carried out in an institutional setting.

Patients with KC, aged from 12 to 30 years old, followed in our Ophthalmology Corneal Department, were identified and consecutively included between December 2017 and February 2018.

All KC patients selected were followed by a corneal specialist (JPC or LT), and all examinations were performed by these two researchers.

Collected data included sex, age, patient's ocular history, medical history (allergy and eye rubbing), and a family history of KC. We have considered the following allergic conditions: allergic asthma, atopic dermatitis, and/or allergic rhinitis. Ophthalmic examination included measurement of the best-corrected Snellen visual acuity (BCVA), slit-lamp biomicroscopic examination, intraocular pressure measurement with Goldmann applanation tonometry, and fundus examination with mydriasis and a 90D lens to exclude other ocular pathologies. The morphological characterization of the cornea was performed using Scheimpflug corneal tomography analysis (Pentacam HR, OCULUS Optikgeräte GmbH, Wetzlar, Germany).

The control group was obtained from our database and included 39 age-matched non-KC subjects.

Exclusion criteria were as follows: existence of active systemic or ocular inflammation; current treatment with systemic or local anti-inflammatory drugs; existence of any other ocular pathology than KC; other ocular surgeries besides intracorneal ring segments or crosslinking procedures (performed at least 6 months prior to the scan); and any other systemic diseases rather than allergic conditions. Subjects whose acquired OCT images were of poor quality were also excluded.

### 2.2. Imaging

**Figure 1:**
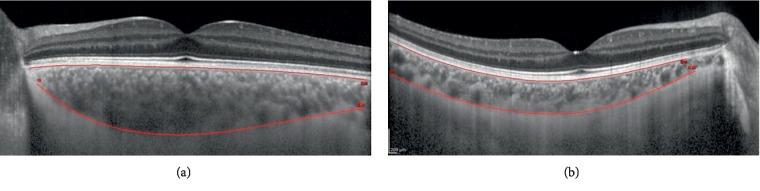
Representation of a choroidal thickness measurement using the semiautomatic mode in a keratoconus (a) and a control eye (b).

The patients underwent EDI SD-OCT using the Spectralis® Heidelberg® apparatus (Heidelberg Engineering, Carlsbad, CA, USA). The SD-OCT scans were single 30-degree. B-scans centered on the fovea using the EDI function averaged 100 times. CT was measured on the horizontal OCT from the outer edge of the hyperreflective line, corresponding to the retinal pigment epithelium, to the choroidal-scleral junction, as illustrated in Figure 1. These measurements were taken at the subfoveal choroid and at 500 *μ*m intervals from the fovea: temporal 500 *μ*m (T500), 1000 *μ*m (T1000), and 1500 *μ*m (T1500) and nasal 500 *μ*m (N500), 1000 *μ*m (N1000), and 1500 *μ*m (N1500).

CT measurements were performed manually by two masked independent observers (JVP and JB). Changes in the thickness in the submacular choroid were analyzed.

### 2.3. Statistical Evaluation

Statistical analysis was performed using the SPSS® statistical software (version 24.0 for Mac OS; SPSS Inc., Chicago, IL., USA). In the present study, both eyes of each subject were used for statistical analyses. The Kolmogorov–Smirnov test and normal probability plots were used to confirm the normal distribution of the data. Parametric or nonparametric tests were used for continuous variables comparison between the keratoconus and control group, according to the normality of data. Chi-squared or Fisher's exact tests were performed for categorical variables comparison. Multivariate linear regression analysis, using generalized linear models, was performed to identify the potential variables associated with subfoveal choroidal thickness. Statistical significance for all the analyses was set at *p* value less than 0.05.

## 3. Results

A total of 212 eyes were included in this study: 134 eyes from 74 KC patients and 78 eyes from 39 controls. A sample characterization of controls and KC groups is presented in Table 1. The mean age was 23.01 ± 4.68 in the study group and 22.40 ± 5.77 in the control group. There was no statistical difference between groups when it comes to age and spherical equivalents, whereas there was a statistical difference when it comes to gender and BCVA (*p* < 0.001). Individuals in the KC group were predominantly men (31.3% women in the KC group), while individuals in the control group were predominantly women (61.5% women in the control group). Best-corrected visual acuity was 0.80 ± 0.21 in the KC group, while in the control group it was 0.98 ± 0.06.

In the KC group, 42 eyes (31.3%) had a documented history of atopy or eye rubbing. The mean BCVA with glasses or contact lenses was 0.8 ± 0.21, taking into consideration that contact lenses were only tried in 85 keratoconus eyes, with 29 eyes not tolerating them. Regarding previous surgeries, 12 eyes (9%) were implanted with an intrastromal corneal ring and crosslinking was performed in 22 eyes (16.4%) in order to stabilize the progression of the disease. A characterization of topographic and pachymetric indices in the KC group is listed in Table 2. In this group, the mean *K*_max_ was 56.49 ± 7.83, the mean ISV was 91.55 ± 40.94, the mean PAK min was 456.66 ± 51.91, the mean Bad-D was 9.25 ± 5.23 and the median keratoconus classification was 2.5.

**Figure 2:**
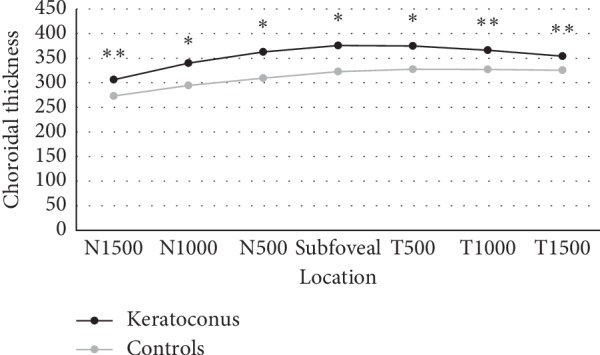
Mean choroidal thickness in each group along a horizontal section, measured in microns. ^*∗*^*p* < 0.001; ^*∗∗*^*p* < 0.005.

We found significant statistically thicker choroids in every measured location, as shown in Table 3 and Figure 2. Subfoveal mean CT values obtained were 375.86 ± 89.29 and 322.91 ± 85.14 in keratoconus and control groups, respectively (*p* < 0.001).

We analyzed which variables could be considered covariates and influence CT. In the keratoconus group, there was no statistical difference in CT on any measured location between different genders, between the presence or absence of risk factors, or between KC patients who underwent intracorneal ring or crosslinking and KC patients who were not subjected to any kind of surgery (all *p* > 0.05 in every location using the Mann–Whitney *U* test). In a multivariate analysis, CT was significantly associated with spherical equivalent (*p*=0.004) and the presence of keratoconus (*p* < 0.001), but not with age (*p*=0.167), gender (*p*=0.579), or BCVA (*p*=0.178). In a “fixed model,” subfoveal CT was found to significantly increase on average 8.48 *μ*m (95% CI +2.74 to +14.23) for each increase of one diopter of spherical equivalent; however, keratoconus patients were found to have a 67.55 *μ*m (95% CI 36.61 to 98.49) thicker choroid compared to matched controls.

## 4. Discussion

The choroid has been implicated in the pathogenesis of many inflammatory disorders of the eye. Recent studies using EDI SD-OCT, an enhanced image from Bruch's membrane to the suprachoroidal space, have shown anatomical choroidal changes in various inflammatory eye disorders and in multisystemic inflammatory disorders without ophthalmologic manifestations [26, 31–37]. These findings may support a choroidal role in the pathogenesis of these inflammatory disorders. Recent data also show the existence of an inflammatory microenvironment in KC patients [13, 14]. Taking this into account, in this study, we aimed to analyze CT using EDI SD-OCT scans in KC eyes and compare them to healthy controls.

Our results showed a thicker choroid in KC eyes compared with healthy eyes in every measured location, with KC patients showing a 67.55 *μ*m (95% CI 36.61 to 98.49) thicker subfoveal choroid compared to matched controls. Our control group (mean age 22.40 ± 5.77) has a mean subfoveal CT of 322.91 ± 85.14 *μ*m, similarly to the results reported by Tan et al. in a population with a similar age [38].

Gutierrez-Bonet et al., in a recently published study, presented similar results with swept-source OCT, showing that CT in KC patients is statistically thicker than that in healthy population (an average increase in CT of 34%), which corroborates our findings [27]. However, this difference was less evident in older subjects (>45 years, nonsignificant increase of 7%, *P*=0.37) and largest in younger age groups (<25 years, average increase of 40%, *p* < 0.001) [27].

It is known that spherical equivalent and age influence CT. Thus, we performed a multivariate analysis adjusted to covariates to analyze which variables could influence our results. Our results confirm previous findings that CT is significantly associated with spherical equivalent (*p*=0.004). Despite knowing that CT varies inversely with age, our results did not show an association between age and CT, which could be due to the young and small age interval in both study and control groups. Furthermore, it was shown that subfoveal CT was negatively correlated with age only in older patients, where no significant correlation was shown before the fifth decade of life [39, 40], and it was found that there was no difference between subfoveal choroidal thickness in the pediatric population (10 ± 3 years) when compared to an adult population (53 ± 16 years) [41].

The exact pathophysiological mechanism resulting in a thicker choroid in KC patients is not known, but we speculate that this could possibly be associated with inflammatory choroidal mechanisms in keratoconic eyes. Despite KC being classically defined as a noninflammatory disease [1, 2] and the fact that no consistent relation to systemic inflammatory disorders has been described, a role for inflammation in the pathophysiology of KC has been suggested.

In fact, a large number of studies provided evidence of increased levels of proinflammatory cells, cytokines, and other inflammatory mediators in tears of KC patients, whereas other inflammatory suppressants seem to be reduced [12–14]. Multiple inflammatory mediators have been found to be increased in tears of KC patients, including the well-documented IL-1, IL-6, TNF-a, and MMP-9 [15–17]. These inflammatory mediators operate actively at the ocular surface and affect the corneal microenvironment, which could play an important role in the KC pathogenesis [12, 13]. Furthermore, Arnal et al. described increased levels of oxidative stress markers and decreased antioxidant capacity in KC corneas, which might be involved in the development of the disease [42].

In addition to local activation of inflammatory pathways, there is accumulating evidence that systemic inflammatory changes and systemic oxidative stress may affect the corneal microenvironment in KC [12, 18]. For example, systemic inflammation monitored via the neutrophil-to-lymphocyte ratio was recently associated with progressive KC [43], and systemic oxidative stress has been reported in KC patients [18]. Increased frequency of neutrophils indicates proinflammatory conditions, and neutrophils are associated with the activation of MMPs, which have been found to be elevated in KC [43]. Moreover, systemic inflammatory markers such as elevated toll-like receptor- (TLR-) 2 and 4 expression in neutrophils and monocytes and higher serum levels of interleukin- (IL-) 1*β*, IL-6, tumor necrosis factor- (TNF-) *α*, MMP-9, and NF-*κ*B were found in KC patients [19]. Furthermore, vitamin D serum levels were found to be lower in patients with KC when compared with age- and sex-matched healthy controls, even though there was no association with KC severity. Vitamin D is known to modulate both the innate and adaptive immune systems and to suppress allergic pathways, with many studies linking vitamin D deficiency with autoimmune diseases and allergic disorders [44].

Cristina Kenney et al. proposed a “cascade hypothesis” where keratoconus corneas could have abnormal or defective enzymes in the lipid peroxidation and/or nitric oxide pathways, leading to oxidative damage. The accumulation of oxidative and cytotoxic products would cause an alteration in various corneal proteins, triggering a cascade of events (i.e., apoptosis, altered signaling pathways, increased enzyme activities, and fibrosis) [45]. On the other hand, Galvis et al. suggested that the pathophysiology of KC could have various events occurring simultaneously which could present positive feedback among each other [13]. Apart from the pathophysiological process, an inflammatory basis to KC appears to be a growing concordance. Being the choroid a vascular cushion of the eye, its increased thickness in KC eyes may be one more evidence supporting an inflammatory ill condition in such patients.

On the other hand, inflammation is not the only possible explanation to changes in the choroid. It is known that many vascular conditions may alter the morphology of the choroid, whereas diabetic retinopathy is one of the most studied conditions. Even though there is some disagreement about CT in patients with diabetes, some studies show a thicker choroid in patients with diabetic retinopathy, mainly in patients with mild nonprogressive retinopathy or untreated retinopathy. Still, the explanation for this finding remains unknown [46]. Furthermore, in patients with central serous chorioretinopathy (CSCR), increased choroidal thickness was linked to choroidal capillary hyperpermeability. Indocyanine green angiography shows choroidal hyperpermeability, irregular dilation of choroidal veins, and choroidal lobular ischemia, and EDI SD-OCT shows thickening of the choroid, which further supports the idea of vascular congestion and elevated hydrostatic pressure in these patients. Although active CSCR has been correlated with elevated levels of endogenous and exogenous corticosteroids, the actual cause is still in speculation [47].

The cause of an increased CT in KC eyes is not known, but it may be associated with inflammatory pathways and increased choroidal blood flow and permeability. Furthermore, longitudinal studies may help clarify the role of these findings.

If the finding of a thicker choroid in keratoconic eyes is only an association without any role in the disease, or if it plays a part in the pathogenesis process is unknown, but another important clue was raised by Gutierrez-Bonet et al. when they observed that the increased choroidal thickness is only found till age 45. While KC patients aged less than 45 showed statistically thicker choroids when compared to healthy controls, those aged older than 45 did not [27]. KC typically progresses over a period of 15 to 20 years from its diagnosis, usually during early adult life. Theories that claim a role for inflammation in the pathophysiology of KC might help explain this course of events, supporting the idea that inflammatory factors could potentially contribute to both KC activity and increase of CT [27].

A limitation of this study is its cross-sectional design, which clouds the determination of a causal relationship between the altered choroid profile and KC development. Moreover, a longitudinal study could control better some possible biases of this single point observation.

Another limitation of this study is that we have only used the horizontal OCT and we did not use the vertical OCT to calculate CT. In addition to this, some characteristics, such as gender and atopy, were different in both groups. Even though there was no association between these characteristics and CT in a multivariate analysis, these could still influence the results. Furthermore, in this study, we included some patients who underwent intracorneal ring segments or crosslinking procedures in the past; although they were performed at least 6 months prior to the scan and that we did not find any difference between KC patients who underwent this kind of surgeries and patients who did not, these procedures could have some influence in CT.

In conclusion, keratoconic eyes seem to have a thicker choroid compared with healthy eyes, which could be associated with inflammatory choroidal mechanisms in these patients. Whether this finding is only an association without any role in this disease, or whether it plays a part in the pathogenesis process is unknown. Longitudinal studies will help to better understand whether there is any causality between eventual changes that occur in the choroid and the pathways of the ectactic cornea. Furthermore, the correlation between KC progression and CT or if atopy could play a role in CT in these patients is yet to be studied. Further research is needed in order to better understand the role of CT in this disease and whether EDI SD-OCT could be used as a tool to better assess KC patients.

## Figures and Tables

**Table 1:** Summary of demographical and clinical characteristics by group.

	Keratoconus (*n* = 134)	Controls (*n* = 78)	*p*
Age, years	23.01 ± 4.68	22.40 ± 5.77	*p*=0.364
Spherical equivalent, diopters	−2.19 ± 2.24	−1.94 ± 1.94	*p*=0.409
BCVA, decimal^a^	0.80 ± 0.21	0.98 ± 0.06	*p* < 0.001
Women, *n*	*n* = 42 (31.3%)	*n* = 48 (61.5%)	*p* < 0.001

Results are expressed as mean ± SD for continuous variables, and gender is expressed as count and percentage of women. BCVA, best-corrected visual acuity.

**Table 2:** Characterization of topographic and pachymetric indices in the keratoconus group.

Variables	Values
*K*_max_	56.49 ± 7.83
Kmed	48.50 ± 5.40
K2	50.19 ± 6.00
Keratoconus classification	2.5 (0–4)
ISV	91.55 ± 40.94
PAK min	456.66 ± 51.91
ART max	170.73 ± 100.07
Bad-D	9.25 ± 5.23

Results are expressed as mean ± SD for continuous variables and median (range) for keratoconus classification.

**Table 3:** Choroidal thickness in different locations by group.

	Keratoconus (*N* = 134)	Controls (*n* = 78)	*p*
	Median (range)	Median (range)	
Subfoveal	371.50 (193–659)	301 (174–500)	*p* < 0.001
N500	364 (174–638)	291 (176–509)	*p* < 0.001
N1000	336 (163–623)	276 (182–509)	*p* < 0.001
N1500	293 (108–595)	254 (164–494)	*p* < 0.006
T500	369 (210–644)	305 (185–512)	*p* < 0.001
T1000	374 (180–622)	311.50 (192–507)	*p* < 0.001
T1500	358 (167–596)	307 (210–493)	*p*=0.014

Results are expressed as median (range). Measurements undertaken at subfoveal, temporal 500 *μ*m (T500), 1000 *μ*m (T1000), and 1500 *μ*m (T1500), and nasal 500 *μ*m (N500), 1000 *μ*m (N1000), and 1500 *μ*m (N1500).

## Data Availability

The data used to support the findings of this study are available from the corresponding author upon request.

## References

[1] Krachmer J. H., Feder R. S., Belin M. W. (1984). Keratoconus and related noninflammatory corneal thinning disorders. *Survey of Ophthalmology*.

[2] Rabinowitz Y. S. (1998). Keratoconus. *Survey of Ophthalmology*.

[3] Khaled M. L., Helwa I., Drewry M., Seremwe M., Estes A., Liu Y. (2017). Molecular and histopathological changes associated with keratoconus. *BioMed Research International*.

[4] Godefrooij D. A., Mangen M.-J. J., Chan E. (2017). Cost-effectiveness analysis of corneal collagen crosslinking for progressive keratoconus. *Ophthalmology*.

[5] Gokhale N. (2013). Epidemiology of keratoconus. *Indian Journal of Ophthalmology*.

[6] Gordon-Shaag A., Millodot M., Shneor E., Liu Y. (2015). The genetic and environmental factors for keratoconus. *BioMed Research International*.

[7] Naderan M., Shoar S., Rezagholizadeh F., Zolfaghari M., Naderan M. (2015). Characteristics and associations of keratoconus patients. *Contact Lens and Anterior Eye*.

[8] Weed K. H., MacEwen C. J., Giles T., Low J., McGhee C. N. J. (2008). The Dundee University Scottish Keratoconus study: demographics, corneal signs, associated diseases, and eye rubbing. *Eye*.

[9] Millodot M., Shneor E., Albou S., Atlani E., Gordon-Shaag A. (2011). Prevalence and associated factors of keratoconus in Jerusalem: a cross-sectional study. *Ophthalmic Epidemiology*.

[10] Harrison R. J., Klouda P. T., Easty D. L., Manku M., Charles J., Stewart C. M. (1989). Association between keratoconus and atopy. *British Journal of Ophthalmology*.

[11] Karolak J. A., Gajecka M. (2017). Genomic strategies to understand causes of keratoconus. *Molecular Genetics and Genomics*.

[12] Wisse R. P. L., Kuiper J. J. W., Gans R., Imhof S., Radstake T. R. D. J., Van der Lelij A. (2015). Cytokine expression in keratoconus and its corneal microenvironment: a systematic review. *The Ocular Surface*.

[13] Galvis V., Sherwin T., Tello A., Merayo J., Barrera R., Acera A. (2015). Keratoconus: an inflammatory disorder?. *Eye*.

[14] Pasztor D., Kolozsvari B. L., Csutak A. (2016). Scheimpflug imaging parameters associated with tear mediators and bronchial asthma in keratoconus. *Journal of Ophthalmology*.

[15] Lema I., Duran J. (2005). Inflammatory molecules in the tears of patients with keratoconus. *Ophthalmology*.

[16] Lema I., Sobrino T., Duran J. A., Brea D., Diez-Feijoo E. (2009). Subclinical keratoconus and inflammatory molecules from tears. *British Journal of Ophthalmology*.

[17] Sorkhabi R., Ghorbanihaghjo A., Taheri N., Ahoor M. H. (2015). Tear film inflammatory mediators in patients with keratoconus. *International Ophthalmology*.

[18] Toprak I., Kucukatay V., Yildirim C., Kilic-Toprak E., Kilic-Erkek O. (2014). Increased systemic oxidative stress in patients with keratoconus. *Eye*.

[19] Sobrino T., Regueiro U., Malfeito M. (2017). Higher expression of toll-like receptors 2 and 4 in blood cells of keratoconus patiens. *Scientific Reports*.

[20] Shetty R., Ghosh A., Lim R. R. (2015). Elevated expression of matrix metalloproteinase-9 and inflammatory cytokines in keratoconus patients is inhibited by cyclosporine A. *Investigative Ophthalmology & Visual Science*.

[21] Maruko I., Iida T., Sugano Y. (2011). Subfoveal choroidal thickness after treatment of Vogt-Koyanagi-Harada disease. *Retina*.

[22] Kim M., Kim H., Kwon H. J., Kim S. S., Koh H. J., Lee S. C. (2013). Choroidal thickness in Behcet’s uveitis: an enhanced depth imaging-optical coherence tomography and its association with angiographic changes. *Investigative Opthalmology & Visual Science*.

[23] Fleischman D., Say E. A. T., Wright J. D., Landers M. B. (2012). Multimodality diagnostic imaging in a case of sympathetic ophthalmia. *Ocular Immunology and Inflammation*.

[24] Hirukawa K., Keino H., Watanabe T., Okada A. A. (2013). Enhanced depth imaging optical coherence tomography of the choroid in new-onset acute posterior scleritis. *Graefe’s Archive for Clinical and Experimental Ophthalmology*.

[25] Baltmr A., Lightman S., Tomkins-Netzer O. (2014). Examining the choroid in ocular inflammation: a focus on enhanced depth imaging. *Journal of Ophthalmology*.

[26] Ferreira C. S., Beato J., Falcão M. S., Brandão E., Falcão-Reis F., Carneiro Â. M. (2017). Choroidal thickness in multisystemic autoimmune diseases without ophthalmologic manifestations. *Retina*.

[27] Gutierrez-Bonet R., Ruiz-Medrano J., Peña-Garcia P. (2018). Macular choroidal thickening in keratoconus patients: swept-source optical coherence tomography study. *Translational Vision Science & Technology*.

[28] Akkaya S. (2018). Macular and peripapillary choroidal thickness in patients with keratoconus. *Ophthalmic Surgery, Lasers and Imaging Retina*.

[29] Yilmaz I., Saracoglu Yilmaz B., Guleryuz N. B., Perente I., Ozkaya A., Taskapili M. (2018). Assessment of the macula and choroid in pediatric keratoconus patients. *Saudi Journal of Ophthalmology*.

[30] Sezer T., Altınışık M., Koytak İ. A., Özdemir M. H. (2016). The choroid and optical coherence tomography. *Türk Oftalmoloji Dergisi*.

[31] Brito P. N., Rosas V. M., Coentrão L. M. (2015). Evaluation of visual acuity, macular status, and subfoveal choroidal thickness changes after cataract surgery in eyes with diabetic retinopathy. *Retina*.

[32] Chung S. E., Kang S. W., Lee J. H., Kim Y. T. (2011). Choroidal thickness in polypoidal choroidal vasculopathy and exudative age-related macular degeneration. *Ophthalmology*.

[33] Falcao M. S., Goncalves N. M., Freitas-Costa P. (2013). Choroidal and macular thickness changes induced by cataract surgery. *Clin Ophthalmol*.

[34] Fujiwara T., Imamura Y., Margolis R., Slakter J. S., Spaide R. F. (2009). Enhanced depth imaging optical coherence tomography of the choroid in highly myopic eyes. *American Journal of Ophthalmology*.

[35] Iaccarino G., Cennamo G., Forte R., Cennamo G. (2009). Evaluation of posterior Pole with echography and optical coherence tomography in patients with behçet’s disease. *Ophthalmologica*.

[36] Read R. W., Rao N. A., Cunningham E. T. (2000). Vogt-Koyanagi-Harada disease. *Current Opinion in Ophthalmology*.

[37] Ross A., Ross A. H., Mohamed Q. (2011). Review and update of central serous chorioretinopathy. *Current Opinion in Ophthalmology*.

[38] Tan C. S. H., Cheong K. X., Lim L. W., Li K. Z. (2014). Topographic variation of choroidal and retinal thicknesses at the macula in healthy adults. *British Journal of Ophthalmology*.

[39] Ruiz-Medrano J., Flores-Moreno I., Peña-García P., Montero J. A., Duker J. S., Ruiz-Moreno J. M. (2014). Macular choroidal thickness profile in a healthy population measured by swept-source optical coherence tomography. *Investigative Opthalmology & Visual Science*.

[40] Ding X., Li J., Zeng J. (2011). Choroidal thickness in healthy Chinese subjects. *Investigative Opthalmology & Visual Science*.

[41] Ruiz-Moreno J. M., Flores-Moreno I., Lugo F., Ruiz-Medrano J., Montero J. A., Akiba M. (2013). Macular choroidal thickness in normal pediatric population measured by swept-source optical coherence tomography. *Investigative Opthalmology & Visual Science*.

[42] Arnal E., Peris-Martínez C., Menezo J. L., Johnsen-Soriano S., Romero F. J. (2011). Oxidative stress in keratoconus?. *Investigative Opthalmology & Visual Science*.

[43] Karaca E. E., Özmen M. C., Ekici F., Yüksel E., Türkoğlu Z. (2014). Neutrophil-to-lymphocyte ratio may predict progression in patients with keratoconus. *Cornea*.

[44] Akkaya S., Ulusoy D. M. (2019). Serum vitamin D levels in patients with keratoconus. *Ocular Immunology and Inflammation*.

[45] Cristina Kenney M., Brown D. J. (2003). The cascade hypothesis of keratoconus. *Contact Lens and Anterior Eye*.

[46] Melancia D., Vicente A., Cunha J. P., Abegão Pinto L., Ferreira J. (2016). Diabetic choroidopathy: a review of the current literature. *Graefe’s Archive for Clinical and Experimental Ophthalmology*.

[47] Maruko I., Iida T., Sugano Y., Ojima A., Sekiryu T. (2011). Subfoveal choroidal thickness in fellow eyes of patients with central serous chorioretinopathy. *Retina*.

